# Complete Genome Sequence of the Neonatal Meningitis-Causing *Escherichia coli* Strain NMEC O18

**DOI:** 10.1128/genomeA.01239-16

**Published:** 2016-11-03

**Authors:** Bryon A. Nicholson, Yvonne M. Wannemuehler, Catherine M. Logue, Ganwu Li, Lisa K. Nolan

**Affiliations:** a Department of Veterinary Microbiology and Preventive Medicine, College of Veterinary Medicine, Iowa State University, Ames, Iowa, USA; b Department of Veterinary Diagnostic and Production Animal Medicine, College of Veterinary Medicine, Iowa State University, Ames, Iowa, USA

## Abstract

Neonatal meningitis *Escherichia coli* (NMEC) is a common agent of neonatal bacterial meningitis, causing high neonatal mortality and neurologic sequelae in its victims. Here, we present the complete genome sequence of NMEC O18 (also known as NMEC 58), a highly virulent (O18ac:K1, ST416) strain.

## GENOME ANNOUNCEMENT

Neonatal bacterial meningitis (NBM) is a devastating disease of newborns that can occur during the first four weeks of life (1, 2). Neonatal meningitis *Escherichia coli* (NMEC) is the second-leading cause of NBM (1); it predominantly infects newborns after four days of life (2), but can cause disease in newborns less than one day of age (2). NBM rapidly progresses in the infant, with empirical treatment often performed before a definitive diagnosis of meningitis is reached (3). Reported mortality rates vary from 10% to 50%, based on world region, gestational age, and weight of the neonate (2, 4). Survivors of NBM often suffer severe neurological sequelae that may include seizures, hearing loss, cerebral palsy, and delayed development, occurring in 12% to 44% of survivors (5).

NMEC strain O18 has been characterized in the rat model of meningitis. In neonatal rats, NMEC O18 caused 91% mortality at 24 h postinfection, with mortality observed as early as 14 h (6). NMEC O18 shares the same serogroup and phylogenetic group as the prototypic NMEC strain, RS218 (7, 8). Unlike NMEC RS218, NMEC O18 contains additional genes encoding aerobactin synthesis as well as the outer membrane proteins OmpTp, HlyF, and Iss, but lacks the gene-encoding cytotoxic necrotizing factor 1 found in NMEC RS218 (9, 10). Similar to RS218, NMEC O18 also harbors a large virulence plasmid-carrying genes encoding multiple siderophores and other virulence factors. NMEC O18’s high degree of virulence, despite deviating from the well-described pattern of virulence factors found in NMEC RS218, makes NMEC O18 an interesting candidate for future studies into NMEC pathogenesis.

NMEC O18 is a member of the O18:K1 serotype, the B2 phylogenetic group and the ST416 sequence type (6). NMEC O18 was isolated from the cerebrospinal fluid of an infant with neonatal bacterial meningitis in the Netherlands between 1989 and 1997 (6, 11). NMEC O18 genomic DNA was subjected to genomic sequencing using Roche/454 FLX genome and Illumina HiSeq 2000 sequencers, followed by hybrid assembly. The following data sets were used in the final assembly: (i) GS-FLX, with 456,508 paired reads and 213,580 shotgun reads totaling 128 Mb (~26-fold coverage); and (ii) an Illumina 100-bp paired-end library with 15,458,728 reads totaling 1,236 Mb (~247-fold coverage). Both 454 read sets were assembled *de novo* using Newbler version 2.6 (Roche), and Illumina reads were assembled separately with Velvet version 1.1 (8). The genome was closed using 454 scaffolds against the NMEC strain S88 sequence for Velvet assembly. Whole-genome optical mapping (OpGen, Gaithersburg, MD, USA) was used to validate scaffolds and contig order. The assembly was confirmed using PCR and Sanger sequencing and validated by consistency of paired-end evidence from 454 and Illumina reads.

The assembled genome consists of a single chromosome (5,002,781 bp; 50.75% GC content) and a single plasmid (153,378 bp; 48.49% GC content). The chromosome contains 4,659 protein-encoding genes and 90 tRNA genes.

### Accession number(s).

The whole-genome sequence of NMEC O18 has been deposited in GenBank under the accession number CP007275.
